# Long non-coding RNA TUSC7 suppresses osteosarcoma by targeting miR-211

**DOI:** 10.1042/BSR20190291

**Published:** 2019-11-12

**Authors:** Menglin Cong, Rui Jing

**Affiliations:** 1 Department of Orthopaedic, Qi Lu Hospital of Shandong University, No.107 Wenhua Xi Road, Jinan City 250012, Shandong Province, P.R. China; 2 Department of Radiology, The Second Hospital of Shandong University, No. 247 Beiyuan Road, Jinan City 250033, Shandong Province, P.R. China

**Keywords:** Long non-coding RNAs, MiR-211, osteosarcoma, TUSC7

## Abstract

Long non-coding RNAs (lncRNAs) play a critical role in regulating cancer progression and metastasis. LncRNA tumor suppressor candidate 7 (TUSC-7) was shown to be a tumor suppressor in osteosarcoma. However, the regulation mechanism of TUSC-7 in osteosarcoma is unknown. Bioinformatics analysis showed that TUSC7 specifically binds to miR-211. MiR-211 was up-regulated in osteosarcoma and negatively correlated with the expression of TUSC7. miR-211 expression was inhibited remarkably by TUSC7 overexpression and the reciprocal inhibition exists between TUSC7 and miR-211. RNA pull-down and luciferase reporter assays were used to validate the sequence-specific correlation between miR-211 and TUSC7. TUSC7 inhibited the proliferation, migration of osteosarcoma cells and promoted cellular apoptosis, which is largely mediated by miR-211. We conclude that the TUSC7 acted as a tumor suppressor gene, which is negatively regulated by miR-211. Our study could suggest a potentially novel therapeutic strategy against osteosarcoma.

## Introduction

Osteosarcoma is considered to be the most common primary malignant bone tumor. Osteosarcoma appears mainly in the second to the third decades of life and is a main cause of morbidity as well as mortality in teenagers worldwide [1]. Despite that current treatment strategies of osteosarcoma have achieved a universal cure rate of 65%, therapy of high-stage osteosarcoma still remains a challenge in clinics. There is a continuing interest in seeking new molecular targets associated with osteosarcoma apoptosis, cell growth, invasion, differentiation, and migration to improve the prognosis of patients with osteosarcoma [2].

Long non-coding RNAs (lncRNAs) are promising molecular targets in osteosarcoma. LncRNAs have recently been identified as key modulators of many biological processes in humans [3]. Dysregulated expression of a large array of lncRNAs in cancer has been discovered [3]. LncRNAs act as either suppressors or promoters in various cancers, including osteosarcoma [4,5], ovarian cancer [6], gastric cancer [7], glioma [8], breast cancer [9], colorectal cancer [10], hepatocellular carcinoma [11] etc. Among many lncRNAs investigated, tumor suppressor candidate 7 (TUSC7) was reported as a novel cancer suppressor gene [4,8,12].

Another important class of cancer-regulatory RNAs are microRNAs (miRNAs), which are small non-coding RNAs that mediate a wide range of biological functions [13]. Accumulating evidences suggest that miRNAs interact with lncRNA, imposing an additional level of post-transcriptional regulation [14]. Several studies have investigated the molecular mechanism of miRNAs in governing the progression of osteosarcoma and the role of lncRNA in these processes has gained particular attention [15].

Herein, the aim of the study was to investigate the mechanism by which TUSC-7 functions as a potential tumor suppressor in osteosarcoma. We showed that the lncRNA TUSC7 acted as a tumor suppressor gene, which was negatively regulated by miR-211, an miRNA that was demonstrated to play a critical roles in tumorigenesis of breast cancer [16]. Our result may potentiate the use of TUSC7/miR-211 axis as a novel therapeutic target in osteosarcoma.

## Materials and methods

### Patients and tissue samples

Thirty-two patients with osteosarcoma who were treated at Department of Orthopedic Surgery, Qi Lu Hospital of Shandong University, between 2000 and 2009 were enrolled in the present study. The clinicopathologic variables were retrospectively reviewed based on medical records.

All patients or their advisers provided informed and written consent. The study was approved by the Ethics Committee of Shandong University School of Medicine.

### Cell culture

MG63 and HOS cells were acquired from Cell Bank of Shanghai Biology Institute, Chinese Academy of Science (Shanghai, China). Dulbecco’s modified Eagle medium (DMEM) (Life Technology) was supplemented with 10% fetal bovine serum (FBS, Life Technologies, U.S.A.), 2 mM l-glutamine, and 1% penicillin/streptomycin (Life Technologies) for culture of MG63 and HOS cells, which were maintained at 37°C in a 5% CO_2_ atmosphere.

### RNA extraction and qRT-PCR analysis

Total RNA was isolated with TRIzol reagent (Invitrogen, Carlsbad, CA, U.S.A.) according to the manufacturer’s instructions. Total RNA 500 ng was reversely transcribed in a final volume of 10 μl using random primers and the PrimeScript RT Master Mix (TaKaRa, Dalian, China). qRT-PCR was performed using the PrimeScript RT reagent Kit and SYBR Premix Ex Taq (TaKaRa, Dalian, China). GAPDH was used as an internal control. The PCR primers used were as follows: TUSC7 sense, 5′-GGAAACAGAAGGCACCTCA-3′ and reverse, 5′-TCTCAGAGGTCAAACAGGCA-3′; GAPDH sense, 5′-GTCAACGGATTTGGTCTGTATT-3′ and reverse, 5′-AGTCTTCTGGGTGGCAGTGAT-3′; small nuclear RNA U1 sense, 5′- GGGAGATACCATGATCACGAAGGT-3′ and reverse 5′-CCACAAATTATGCAGTCGAGTTTCCC-3′. Primers purchased from Ribobio (Guangzhou, China) were used for miR-211-3p qPCR analysis. The PCR was conducted at 95°C for 30 s and followed by 40 cycles of 95°C for 5 s and 60°C for 34 s in the ABI 7500 Real-Time PCR system (Applied Biosystems, Foster City, CA, U.S.A.). The *C*_t_ value for each sample was calculated with the ΔΔ*C*_t_ method, and expression fold changes were calculated using 2^−ΔΔ*C*_T_^ methods.

### Plasmid construction

*TUSC7* gene (http://www.ncbi.nlm.nih.gov/nuccore/213417754?report = fasta) was synthesized and subcloned into pcDNA3.1 (Invitrogen, Shanghai, China). Ectopic expression of TUSC7 was achieved by using the TUSC7 plasmid transfection and empty pCDNA vector (EV) as a control. The expression level of TUSC7 was detected by qPCR.

### Transfection

All plasmid vectors (TUSC7 and EV) for transfection were extracted by DNA Midiprep or Midiprep kit (Qiagen, Hilden, Germany). HCT116 and SW480 cells were seeded on six-well plate, and transfected with the TUSC7 or EV using Lipofectamine 2000 (Invitrogen, Shanghai, China) according to the manufacturer’s instructions. MiR-211-3p mimic and negative control (NC) were designed and synthesized by Life Technology, followed with transfection by Lipofectamine 2000. Small interfering RNA sequences targeting TUSC7 or NC were transfected using Lipofectamine 2000 according to the manufacturer’s instructions. The siRNA sequences were as follows: siRNA1 5′-CCAGAAAGAUAUCAACAAAUU-3′, siRNA2 5′-GGAGAUAUAUCUUGCUAGAGG-3, siRNA3 5′-AGAUUUCAGUGGUAUUGUAUA-3′. Cells were harvested after 48 h for qRT-PCR as well as Western blot analyses. The miR-211 inhibitor, anti-miR-211, with the sequence of 5′-AGGCGAAGGAUGACAAAGGGAA-3′ was transfected to cells at a dose of 50 nM.

### Cell counting kit-8 assay

Cell proliferation was monitored by the Cell Counting Kit-8 (CCK8) assay (Promega) every 24 h following the manufacturer’s protocol. The transfected cells were plated in 96-well plates (3000 cells/well), and then 10 μl of CCK8 solution was added and incubated for 2 h. Each solution was measured spectrophotometrically at 450 nm.

### 5-Ethynyl-2-deoxyuridine analysis

Cell proliferation was measured using 5-ethynyl-2-deoxyuridine (EdU) labeling/detection kit (Ribobio, Guangzhou, China). Cells were seeded in 96-well plates with the density of 5 × 10^3^ cells/well. At 48 h after the transfection, 50 μM EdU labeling medium was added and incubated for 2 h at 37°C at 5% CO_2_. After treatment with 4% paraformaldehyde and 0.5% Triton X-100, cells were stained with anti-EdU working solution, followed by DAPI staining to label cell nuclei. The percentage of EdU-positive cells was calculated after analyses of fluorescent microscopy. Five fields of view were randomly evaluated for each treatment group.

### Western blotting assay

Cells were lysed using mammalian protein extraction reagent RIPA (Beyotime, China) supplemented with protease inhibitors cocktail (Roche, Switzerland) and PMSF (Roche, Switzerland). Protein concentration was measured with the Bio-Rad protein assay kit. Protein extractions of 50 μg were separated by 12% SDS/polyacrylamide gel electrophoresis (SDS/PAGE), followed by electro-transferring to 0.22 μm nitrocellulose membranes (Sigma–Aldrich, U.S.A.). Primary and secondary antibodies were sequentially added. ECL chromogenic substrate was used to visualize the bands and the intensity of the bands was quantified by densitometry (Quantity One software; Bio-Rad). GAPDH was used as a loading control. GAPDH antibody was purchased from Sigma–Aldrich (U.S.A.), and cleaved-caspase-3 antibody was purchased from CST.

### Luciferase assays

Two binding sites of miR-211-3p were mutated according to miRmap (http://mirmap.ezlab.org), and the mutated sequences of TUSC7 were subcloned into pcDNA3.1 vector, yielding TUSC (mut). 293T cells were seeded in 24-well plates at a density of 2.0 × 10^5^ cells per well, followed by a 24-h culture before transfection. Following this, cells in each well were co-transfected with pMIR-REPORT reporter plasmid, NC or miR-211-3p mimics, TUSC (wt) or TUSC (mut) and 10 ng internal control vector pRL-SV40 (Promega, Madison, WI, U.S.A.). After 24 h, cell lysates were harvested. Firefly and *Renilla* luciferase activities were measured by the Dual-Luciferase Reporter Assay System (Promega). The value of relative luciferase activity indicates the firefly luciferase activity normalized to that of *Renilla* for each assay.

### Apoptosis

Apoptosis was assessed using dual-color flow cytometry. Cells were harvested, and apoptosis was detected with Annexin V-FITC apoptosis detection kit (KeyGEN, China) following the manufacturer’s protocol. Flow cytometry (BD, U.S.A.) and CELLQuest 3.0 software (BD, U.S.A.) were used to analyze the data.

### Cell migration assay

*In vitro* migration of MG63 and HOS cells was assayed using Transwell chamber (Costar, Corning, NY, U.S.A.) with polycarbonicmembrane (6.5 mm in diameter, 8 mm pore size). Briefly, the transfected cells were suspended in 100 ml serum-free medium at a density of 5 × 10^5^ cells/ml and added in the upper chamber. In the lower chamber, 600 μl of DMEM/high-glucose or DMEM/F12 medium supplemented with 10% FBS was added. After incubation for 48 h, the cells on the upper membrane surface were mechanically removed. Methanol was used to fix cells that had migrated to or invaded the lower side of the membrane. Twenty percent Giemsa was then used to stain the cells. A microscope was used to count the stained cells, using five randomly chosen fields. The average number of cells was calculated.

### Statistical analysis

Student’s *t* test (two-tailed), one-way ANOVA, Kaplan–Meier curve and Cox-regression were performed to analyze the data using SPSS 18.0 software. *P*-values less than 0.05 were considered as statistically significant.

## Results

### Expression of miR-211 in 35 osteosarcoma patients compared with that in non-tumor tissues

**Figure 1:**
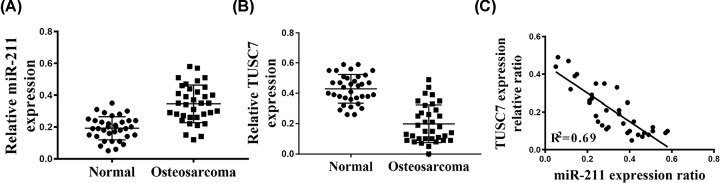
Expression of miR-211 in 35 osteosarcoma patients compared with non-tumor tissues Comparing differences in the expression levels of miR-211 (**A**) and TUSC7 (**B**) between osteosarcoma and non-tumor tissue; *P<0.05, *n*=35. (**C**) TUSC7 expression was negatively correlated with miR-211 expression in osteosarcoma (linear correlation analysis, *r^2^* = 0.69, *n*=35).

We first analyzed the expression level of miR-211 in 35 pairs of osteosarcoma tissues and paired adjacent non-tumor tissues using qRT-PCR analysis. After normalization to paired non-tumor tissues, miR-211 was shown to be significantly up-regulated in osteosarcoma tissues (Figure 1A). We then evaluated the correlation between TUSC7 and miR-211 expression in human samples and found that TUSC7 was significantly down-regulated in osteosarcoma tissue (Figure 1B). Spearman correlation analysis indicated that miR-211 was inversely correlated with TUSC7 expression in human osteosarcoma samples (Figure 1C).

### miR-211 inhibited apoptosis and induced proliferation in MG63 and HOS cell lines

**Figure 2:**
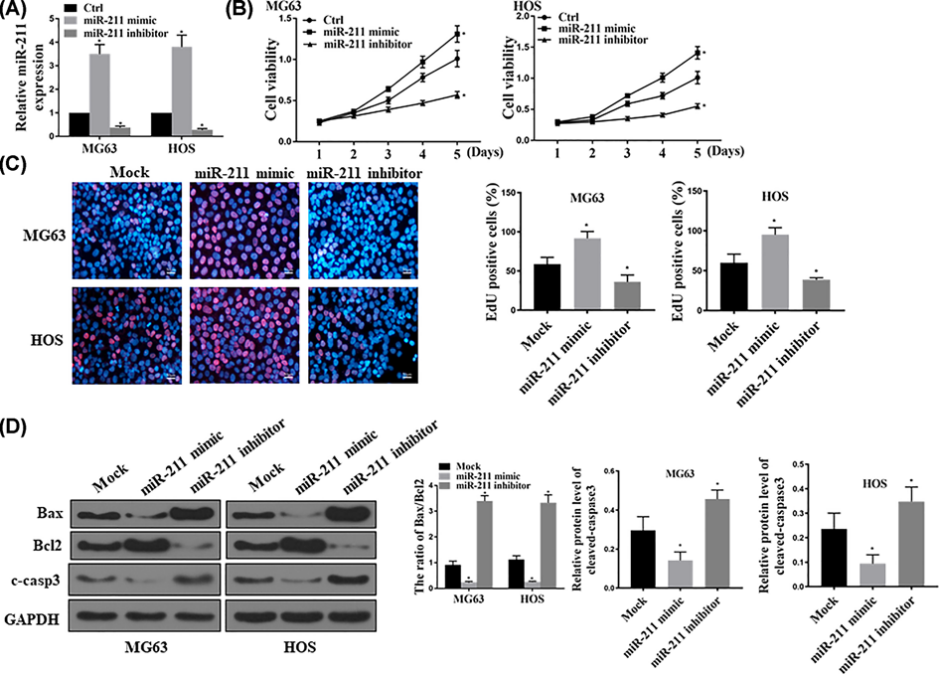
miR-211 inhibited apoptosis and induced proliferation in MG63 and HOS cell lines (**A**) Relative miR-211 expression analyzed by qRT-PCR assay for demonstrating miR-211 overexpression and inhibition by miR-211 mimic and miR-211 inhibitor transfection respectively. (**B**) CCK-8 proliferation assay showing the effects of miR-211 overexpression or inhibition on MG63 and HOS cell proliferation. (**C**) EdU assay showed that ectopic expression of miR-211 affected the percentage of viable cells (MG63 and HOS cells). *P<0.05, *n*=6. (**D**) Western blot assay of cell apoptosis markers, including Bax, Bcl2 and c-casp3, in both MG63 and HOS cell lines. *P<0.05, *n*=5. GAPDH was used as a loading control.

To elucidate the function of miR-211 in osteosarcoma progression, we increased and decreased its expression in MG63 and HOS cell lines through transfection with miR-211 mimic and corresponding inhibitor, which was validated using qRT-PCR analysis of miR-211 levels (Figure 2A). As a result, miR-211 mimic transfection was shown to promote cell proliferation in both MG64 and HOS cells, while miR-211 exerted the opposing effects (Figure 2B). EdU assay revealed that delivery of miR-211 mimic significantly induced the proliferation of MG63 and HOS cells after being cultured for 48 h. miR-211 inhibitor reduced the proliferation comparing with transfection with empty vector (*P*<0.05; Figure 2C). Next, we examined the effect of miR-211 on regulating apoptosis of osteosarcoma. It was shown that the level of cleaved-caspase-3 was high in cells treated with miR-211-inhibitor and low in cells treated with miR-211 mimic (*P*<0.05; Figure 2D).

### TUSC7 increased apoptosis and inhibited proliferation in MG63 and HOS cell lines

**Figure 3:**
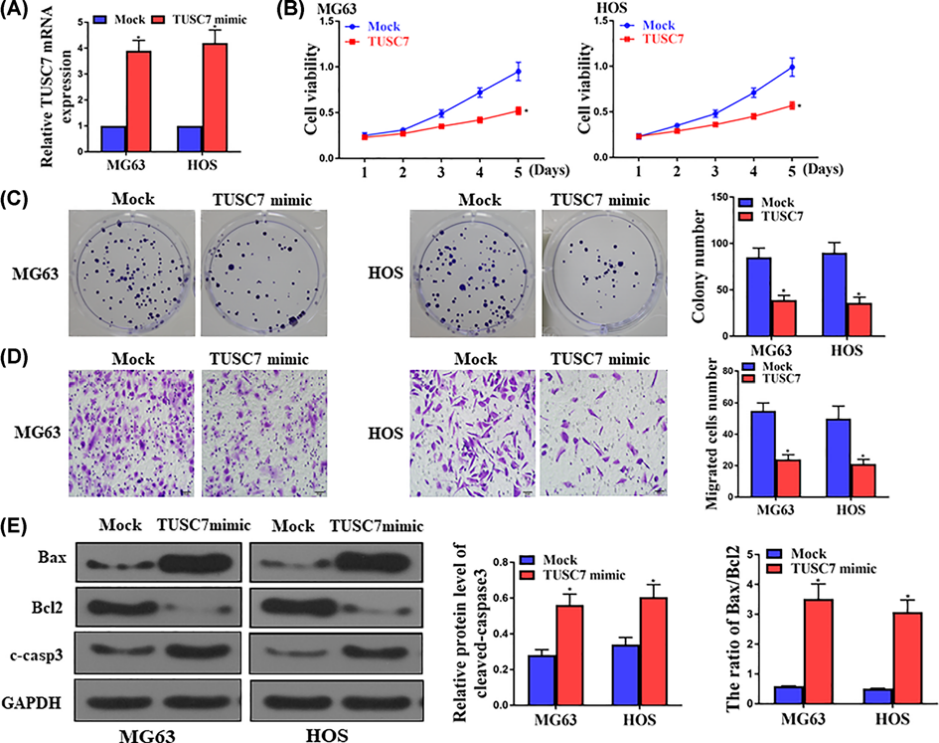
TUSC7 increased apoptosis and inhibited proliferation in MG63 and HOS cell lines ((**A**), MG63 cells) TUSC7 is overexpressed in osteosarcoma cells. (**B**) CCK8 assay showed that ectopic expression of TUSC7 significantly inhibited proliferation rate of osteosarcoma cells. *P<0.05, *n*=5 or 6. (**C,D**) Colony formation and invasion of MG63 and HOS cells with transfection of Mock or TUSC7 mimic. (**E**) Western blot assay in both MG63 and HOS cell lines. *P<0.05, *n*=5.

The levels of TUSC7 after TUSC7 overexpression in MG63 and HOS cells were shown in Figure 3A. CCK8 assay demonstrated that overexpression of TUSC7 significantly inhibited cell proliferation in both MG63 and HOS cells (Figure 3B). Western blot was used to determine the effect of TUSC7 overexpression on cell apoptosis. The suppressive role of TUSC7 was also revealed by invasion (Figure 3C) and colony formation assay (Figure 3D), whereby TUSC7 overexpression inhibited cell invasion and colony formation. Meanwhile, overexpression and down-regulation of TUSC7 induced and reduced apoptosis, respectively, in both MG63 and HOS cell lines (Figure 3E).

### Reciprocal negative regulation of miR-211 and TUSC7

**Figure 4:**
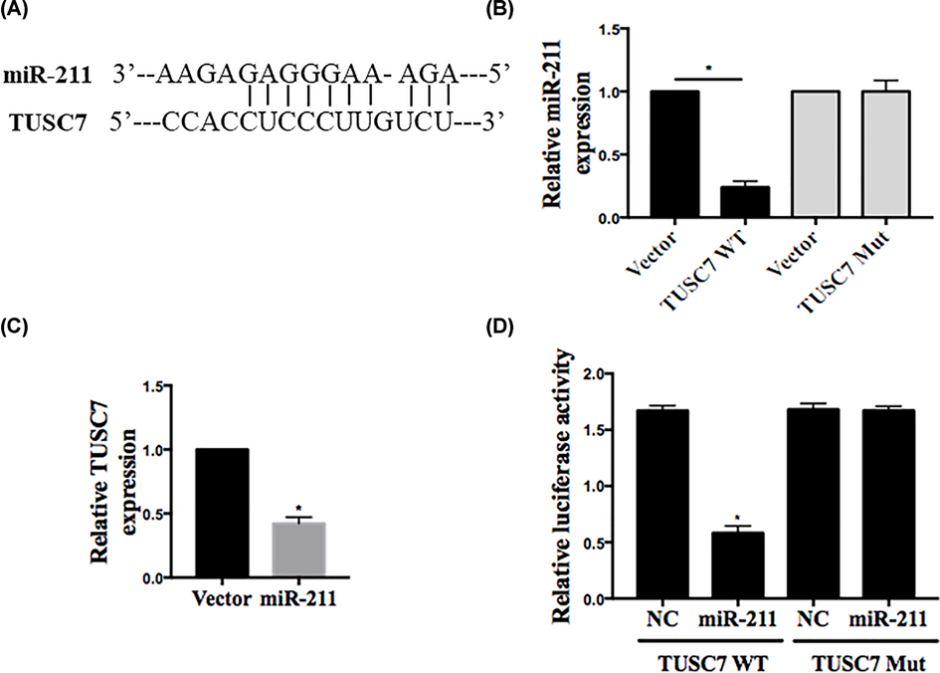
Reciprocal negative regulation of miR-211 and TUSC7 (**A**) Bioinformatics analysis showing the binding site between miR-211 and TUSC7. (**B**) Suppression of miR-211 expression by TUCS7. MG63 cells were first transfected with vector, wild-type TUSC7 (TUSC7-WT) or mutant TUSC7 (TUSC7-mut) with a deletion of two miR-211 binding sites, and 24 h after transfection, total RNA was isolated for qRT-PCR. Error bars represent SD, *n*=6. *P<0.05. (**C**) Effect of miR-211 on TUSC7 expression. MG63 cells were transfected with vector or miR-211, and total RNA was isolated for qRT-PCR 24 h after transfection. Error bars represent SD, *n*=6. *P<0.05. (**D**) The relative luciferase activities were inhibited in the HEK-293 cells co-transfected with wild-type vector and miR-211 mimic, and not with the mutant-type vector. Firefly luciferase activity was normalized to *Renilla* luciferase.

Bioinformatics analysis indicated that a binding site exists between TUSC7 and miR-211 (Figure 4A). We then therefore evaluated whether the level of miR-211 was negatively regulated by TUSC7. In luciferase assay shown in Figure 4B, wild-type TUSC7 expression was reduced by miR-211 overexpression, while mutated TUSC7 was not. As shown in Figure 4B,C, ectopic expression of miR-211 reduced the TUSC7 level. Moreover, the dual-luciferase reporter assay indicated that the relative luciferase activity was decreased in HEK293T transfection with wild-type vector via enhanced miR-211 expression, and the foregoing inhibition was recovered by Mut vector. These results suggested that *TUSC7* is a specific target gene of miR-211 (Figure 4D).

### Overexpression of miR-211 partly reversed TUSC7-induced inhibition of proliferation

**Figure 5:**
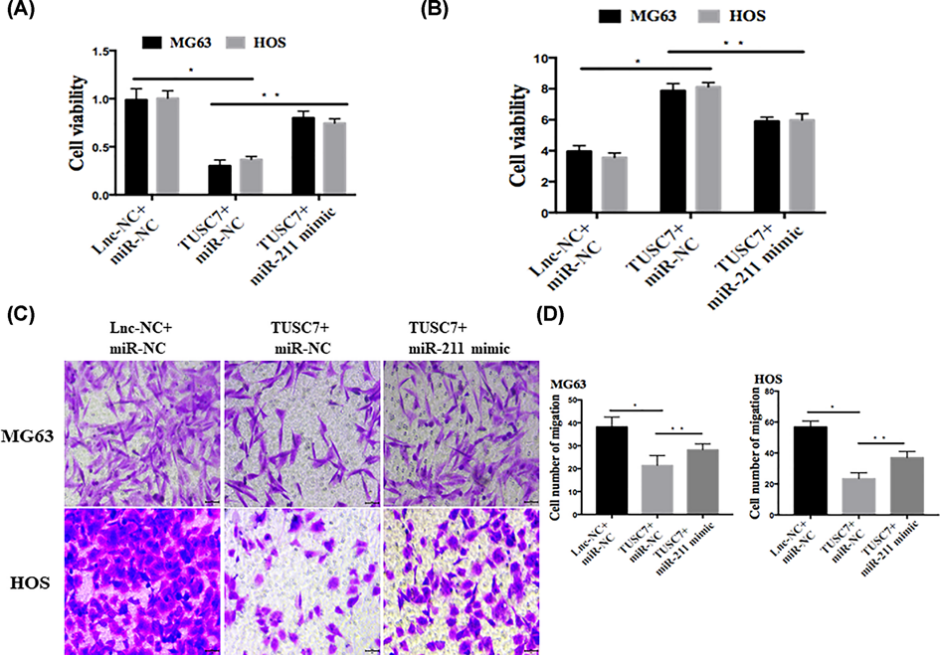
Overexpression of miR-211 partly reversed TUSC7-induced inhibition of proliferation (**A**) Overexpression of miR-211 partly reversed TUSC7-induced inhibition of proliferation. (**B**) Partly reversed TUSC7-induced apoptosis in MG63 and HOS cells. (**C,D**) Migration and invasion in MG63 and HOS cells. *P<0.05, **P<0.05, *n*=5 or 6.

miR-211 mimic was employed to stably transfect into MG63 and HOS cells overexpressing TUSC7. As shown in Figure 5A–D, miR-211 overexpression largely reversed the inhibitory effect of TUSC7 on cell proliferation (Figure 5A,B) and migration (Figure 5C,D). Moreover, overexpression of miR-211 also substantially suppressed cellular apoptosis, despite TUSC7 overexpression (Figure 5B). The tumor suppressive effects of TUSC7 on osteosarcoma were substantially reversed by miR-211 mimic. These results indicated that miR-211 plays an important role in regulating osteosarcoma cells, mediated by TUSC-7.

## Discussions

Osteosarcoma is the most important bone malignant tumor in adolescents, characterized by poor prognosis and high mortality rate [5]. Clinically, the most commonly performed therapeutic methods include surgical resection, chemotherapy and radiotherapy. In recent years, gene therapy, hormonal therapy, immunotherapy and protein therapy have emerged as a new toolset for treatment of osteosarcoma [17]. These alternative therapies are particularly favored in osteosarcoma patients unresponsive to conventional treatments [18]. Here we show that TUSC-7 is a suppressor in osteosarcoma. This result is consistent with previous reports showing that TUSC7 is a novel cancer suppressor gene in many types of human tumors [8]. It was previously shown that TUSC7 low-expression is related to poor prognosis, and increases the proliferation rate of tumor cells [4,19]. In another study, Wang et al. [12] also reported that TUSC7 serves as a tumor suppressor by regulating cancer cell apoptosis, migration, invasion, cell proliferation, cell cycle. TUSC7 overexpression could suppress tumor cell proliferation, invasion, migration and colony formation, suggesting that TUSC7 might be a prognostic and diagnostic biomarker as well as therapeutic target [19].

Our results are in line with previous reports that miR-211 has a strong inhibitive effect on melanoma cell growth, invasion and metastasis [20]. For instance, Bell et al. [21] demonstrated that miR-211 contributes to melanoma adhesion by targeting NUAK1. Inhibition of miR-211 also increases NUAK1 expression and decreases melanoma adhesion [21]. MiR-211 inhibits epithelial-to-mesenchymal transition (EMT) and invasion of cervical cancer cells by targeting mucin protein gene 4, which is up-regulated in several cancers [22]. It was shown that miR-211 plays a significant role in breast cancer metastasis [14]. Also, miR-211 promotes the progression of neck and head carcinomas by targeting transforming growth factor-b type II receptor [23]. It was also shown that miR-211 has important regulatory role in hepatocellular carcinoma metastasis by targeting zinc finger E-box-binding protein ZEB2 [24]. These data orchestrate with our study, underscoring the potential of miR-211 as a therapeutic target in osteosarcoma.

Moreover, the underlying mechanism of TUSC-7 as a potential tumor suppressor is elusive. In the present study, bioinformatics analysis showed that TUSC7 specifically binds to miR-211. MiR-211 was up-regulated in osteosarcoma and negatively correlated with the expression of TUSC7. This result agreed with previous reports showing that TUSC7 was significantly down-regulated in osteosarcoma tissues compared with paired non-tumor tissues [4]. In the present study, the miR-211 expression was inhibited remarkably by the up-regulation of TUSC7 and the reciprocal inhibition was determined between TUSC7 and miR-211. To elucidate that, RNA pull-down and luciferase reporter assays were used to validate the sequence-specific correlation between miR-211 and TUSC7. TUSC7 inhibited the proliferation, migration of osteosarcoma cells and promoted cellular apoptosis largely through miR-211. To date, several miRNAs have been demonstrated to target TUSC7. For example, TUSC7 was shown to inhibit EMT by targeting miR-10a in hepatocellular carcinoma cells [12]. Qi et al. [25] also showed that TUSC7 overexpression negatively regulates miR-23b at post-transcriptional level in gastric cancer cells. Moreover, TUSC7 overexpression restrains cell proliferation, invasion and migration in glioma cells, whereas up-regulated miR-23b reversed the inhibitory effect of TUSC7 (Shang et al. (2016) [8]). However, for osteosarcoma, gene therapy approaches are rarely studied, and further investigations remain to be performed to validate the technology tested in the present study. *In vivo* validations are also needed to translate the findings of the present study into clinics.

## Conclusions

In sum, we reported that miR-211 is up-regulated in osteosarcoma and it demonstrates negative correlation with TUSC7 levels. TUSC7 acts as a tumor suppressor in osteosarcoma. miR-211 and TUSC7 are likely to be promising new therapeutic targets in osteosarcoma.

## Abbreviations

CCK8: cell counting kit-8
CST: Cell Signaling Technology
DMEM: Dulbecco’s modified Eagle medium
EdU: 5-ethynyl-2-deoxyuridine
EMT: epithelial-to-mesenchymal transition
EV: empty pCDNA vector
FBS: fetal bovine serum
lncRNA: long non-coding RNA
miRNA: microRNA
NC: negative control
