# Expression of Concern: Long non-coding RNA TUSC7 suppresses osteosarcoma by targeting miR-211

**DOI:** 10.1042/BSR-2019-0291_EOC

**Published:** 2024-07-31

**Keywords:** Long non-coding RNAs, MiR-211, osteosarcoma, TUSC7

The Editorial Office has been made aware of potential issues surrounding the scientific validity of this paper, hence has issued an expression of concern to notify readers whilst the Editorial Office investigates. It has been noted that Figure 3C MG63 TUSC7 mimic panel in this paper also appears as Figure 1e shRNA-1 panel from Zhang et al, 2020 (DOI: 10.1155/2020/5654380). Additionally, the Western blots appear to have minimal background details and share similarities with other Western blots present in papers by different authors. The authors have been contacted regarding the concerns, but so far have not responded to the Journal.

